# Contribution of Parenting Factors to the Developmental Attainment of 9-Month-Old Infants: Results From the Japan Children’s Study

**DOI:** 10.2188/jea.JE20081014

**Published:** 2009-11-05

**Authors:** Shunyue Cheng, Tadahiko Maeda, Kiyotaka Tomiwa, Noriko Yamakawa, Tatsuya Koeda, Masatoshi Kawai, Tamiko Ogura, Zentaro Yamagata

**Affiliations:** 1 Research Institute of Science and Technology for Society, Japan Science and Technology Agency, Tokyo, Japan; 2 The Institute of Statistical Mathematics, Research Organization of Information and Systems, Tokyo, Japan; 3 Graduate School of Medicine, Kyoto University, Kyoto, Japan; 4 Clinical Research Institute, Mie-chuo Medical Center, National Hospital Organization, Tsu, Japan; 5 Faculty of Regional Sciences, Tottori University, Tottori, Japan; 6 Institute for Education, Mukogawa Women’s University, Nishinomiya, Hyogo, Japan; 7 Graduate School of Humanities, Kobe University, Kobe, Japan; 8 Department of Health Sciences, School of Medicine, University of Yamanashi, Chuo, Yamanashi, Japan

**Keywords:** development, coparenting, maternal stimulation, parenting stress, prospective study

## Abstract

**Background:**

Child development integrates several interdependent domains, but few studies have attempted to identify the common factors that contribute to these different domains of development in infancy. The aim of the present study was to identify the factors that contribute to several domains of developmental attainment in 9-month-old infants.

**Methods:**

We used data from the Japan Children’s Study, a prospective cohort study underway in Japan since 2005. Mothers completed questionnaires about their children’s temperament, coparenting behaviors, maternal parenting stress, and parenting behavior. The Kinder infant development scale was used to evaluate child development outcomes.

**Results:**

A total of 270 children were included in this analysis. After adjusting for the children’s birth weight, gestational age, temperament, and other family environmental variables, multiple logistic regression analyses showed that greater maternal cognitive stimulation was associated with the development of receptive language, expressive language, social relationships, and feeding. Results also suggest that early supportive coparenting helped to promote development in manipulation, receptive language, and social relationships. Maternal parenting stress was stable between the infant ages of 4 and 9 months and was negatively correlated with scores for coparenting and maternal stimulation, which suggests an indirect effect of maternal parenting stress on child outcomes.

**Conclusions:**

Supportive coparenting and maternal cognitive stimulation were the most important contributors to most domains of child development. Our findings suggest that educational interventions targeting young families would help parents establish and maintain an environment of successful coparenting and cognitive stimulation as their children grow.

## INTRODUCTION

Child development is affected by psychosocial, biological, and genetic factors.^1^^,^^2^ Although biological risks are important determinants in all domains of development, psychosocial risks can also adversely affect cognitive and social-emotional competence. The quality of early childhood care has a biological impact on the developing brain, with long-term implications for child development and psychological health.^3^^,^^4^ The Committee on Integrating the Science of Child Development reported that the first few years of life are particularly important because vital development occurs in all domains during this period.^5^

Studies on the association between environmental stimulation and cognition have shown that mothers who were advised to stimulate their babies through a variety of receptive experiences with people, objects, and symbols did indeed contribute to their children’s cognitive development, which resulted in long-term favorable outcomes.^6^ Many researchers agree that maternal schooling has an impact on child development because it provides a structured environment, communicates parental expectations and practices, and offers varied and cognitively stimulating materials for children.^7^

Although mothers are the main caregivers, when fathers spend time with their children, their involvement can assume a broad range of roles and responsibilities. High levels of supportive coparenting and more adaptive family structures are associated with fewer externalizing behavior problems in children.^8^ A longitudinal study suggested that parenting stress in early infancy was associated with behavior problems in toddlers^9^ and preschool children.^10^ However, there is only limited evidence on the effects of early parenting stress and marital quality on child development during the first year.

Child development represents the integration of several interdependent domains, including the motor, language, cognitive, and social-emotional domains. For example, early exploratory movement experiences are important precursors for learning, adaptation, cognition, and socialization,^11^ and developmental speech and language disorders are frequently associated with motor problems.^12^^,^^13^ However, these developmental domains have generally been studied separately and few studies have identified the effects that common explanatory variables have on multiple domains.^14^ In addition, in Japan there have been many studies on the physical health of children, but few on their mental health.

The purpose of this study involving a longitudinal community sample of families was to identify early factors in the family environment—especially parenting factors such as level of maternal stimulation, coparenting behavior, and maternal parenting stress—that contribute to the various domains of infant development at age 9 months.

## METHODS

### Project

The Japan Children’s Study (JCS) project is a prospective developmental cohort study begun in 2005 by the JCS research group at 3 study sites in Japan: Osaka, Mie, and Tottori. The purpose of the project is to describe the development of social skills in children and investigate factors that promote this development. The project includes 2 cohorts: infants and preschoolers. For the infant cohort, the child’s mother and father completed a baseline questionnaire when the infants were 4 months old, to be followed at the ages of 9, 18, 30, and 42 months. The mother–infant dyads also participate in laboratory observations at each measurement occasion.

### Participants

Data from the infant cohort that were collected when the infants were aged 4 and 9 months were used in this study. Participants were recruited at the abovementioned 3 study sites. In Osaka, we recruited children who had visited the Miyakojima Health Care Center for a 3-month infant health checkup, as well as children who were born in the main birth centers in Miyakojima and visited those facilities for a 1-month health examination. In Mie and Tottori, we recruited children who were born and received a 1-month health checkup at 1 of 5 medical facilities (2 hospitals and 1 clinic in Mie and 2 hospitals in Tottori). Infants with non-Japanese mothers, those whose families planned to move out of the area, and those who had serious medical complications were excluded. A total of 935 families (378 from Osaka, 467 from Mie, and 90 from Tottori) were solicited to participate in this cohort and 505 (54%) mothers of infants agreed to participate. Of the 505 families, 479 (95%) mothers with infants born between August 2004 and April 2006 participated in the baseline assessment, when infants were aged 4 months. Mothers completed the self-administered questionnaires sent to them before the 4-month laboratory observation. Among the 479 mother-child dyads, 23 dropped out at 9 months and 456 (95%) dyads continued to participate. Because the project underwent a revision of questionnaires for the baseline assessment, only the 313 mothers (out of 479) who were given, and completed, the final version of the questionnaire were available for analysis. The participants who responded to earlier versions of the questionnaire had similar demographic characteristics at baseline, but were not included in the analysis. Out of these 313 mother-infant pairs, 301 (96%) dyads were followed until infants were aged 9 months, and data from these dyads were analyzed in the present study. At this stage, all participants from the Tottori site were excluded from the analysis. At the baseline assessment there were no significant differences by region in the distributions of the main study variables. A total of 270 mother-infant pairs that had completed both 4- and 9-month questionnaires on all relevant items were available for subsequent statistical analysis.

### Measures

Child development outcomes were assessed using the Kinder Infant Development Scale (KIDS)^15^ when the children were 9 months old. KIDS is a developmental screening scale that is convenient to use and easily completed by parents. It was standardized in 1989 using 6000 Japanese children aged 0 to 6 years. The reliability coefficient of this scale is 0.95, and the correlation coefficient between KIDS and the Stanford-Binet intelligence test is *r* = 0.86; the correlation coefficient between KIDS and the Wechsler Preschool and Primary Scale of Intelligence is *r* = 0.65. Thus, the validity of the scale has been confirmed. In this study, KIDS type A, which was designed for assessing infants aged 1 to 11 months, was used. The scale includes 117 items and is composed of subscales for 6 developmental domains: physical/motor, manipulation, receptive language, expressive language, social relationships with adults, and feeding. Each item has the options of “pass” and “fail.” Responses are scored as 1 or 0 and the scores are summed for each subscale, with a higher score reflecting a higher level of development. The internal consistency, expressed as coefficient alpha, was 0.88. The overall development score ranged between 0.57 and 0.79 for the 6 domains in this sample.

The first measure of family factors—the maternal cognitive stimulation score—was derived from a short-version evaluation of environmental stimulation. Mothers completed the questionnaire when their children were 9 months old. The following items were used in this analysis: “How often do you talk or sing to your baby before bed?”; “How often do you take your baby to the park?”; “How often do you take your baby to visit relations or friends?”; and “When your baby gets a new toy, do you play with your baby?”. Ratings on each item ranged from 1 (rarely) to 4 (almost every day). For the convenience of analysis, the scores were divided into 2 groups: low (scores equal to or below the median) and high (scores above the median) cognitive stimulation.

The second measure of family factors—coparenting behavior—was measured using 3 indicators, all based on the mother’s rating when their infant was 4 months old. The items were: “Do you and your husband agree on child care?”; “Do you and your husband talk about child rearing?”; and “Are you satisfied with your husband’s cooperation?”. Responses were scored 1 (yes) or 0 (no). For the analysis, “no” responses to all 3 items was defined as low supportive coparenting, and a “yes” response to at least 1 item was defined as high supportive coparenting.

A rearing-related stress (RRS) questionnaire^16^ was included to assess maternal parenting stress and administered to mothers when their infants were aged 4 and 9 months. The questionnaire was originally developed to assess the degree of stress experienced by parents in 2 domains: child-RRS and maternal-RRS. The latter consists of 10 items that contribute to maternal stress, for example, “I don’t know how to treat or rear a baby;” “My husband does not pay attention to our baby;” and “I feel confused because there is too much information about child rearing.” There are 4 response categories, from 1 (disagree) to 4 (agree completely), with higher scores indicating higher parenting stress. The Cronbach’s alphas for this sample, calculated separately at each time point, were 0.71 and 0.77, respectively. Mothers who scored higher than the median were defined as having a high level of parenting stress.

When the infants were 9 months old, mothers completed a 42-item questionnaire on child temperament, which was developed for this study.^17^ The selection of the 42 items was based on previous studies.^18^^,^^19^ The questionnaire measures 7 dimensions of temperament: taste reactivity, audiovisual reactivity, negative emotionality, frustration tolerance, persistence in task situations, approach new situations and people, and rhythmicity. Each dimension consists of 6 items. Responses were scored using Likert scale ratings ranging from 1 (almost never) to 6 (almost always) to rate their infant’s standard day-to-day behavior. Internal consistency of these scales was reported by Ogura.^20^ In the analysis, we ultimately used 4 dimensions for which the Cronbach’s alpha was above 0.60: frustration tolerance (0.76), persistence in task situations (0.61), approach new situations and people (0.85), and rhythmicity (0.75); higher scores indicate higher frustration tolerance, higher persistence in task situations, quicker approach to new situations or people, and higher rhythmicity, respectively.

Information on parents’ ages and levels of education, annual family income, type of family, and the children’s sex, birth weight, birth order and gestational age (number of full weeks) was obtained from the questionnaire completed by mothers when the infants were 4 months old.

### Statistical analysis

To describe the demographic characteristic of the subjects, we examined the frequency distributions of independent variables by using response percentages for categorical variables, and medians and ranges for continuous variables. We also examined bivariate associations between exposure variables and outcomes by using the chi-square test to compare the percentage of infants passing the developmental norm for each independent variable with the categories of exposure variables. For continuous variables (eg, infant temperament), Pearson product-moment correlations were computed between each domain of development score and temperament scale scores. Bivariate correlations were also calculated among selected independent variables.

To evaluate the simultaneous contribution of family environment and child factors in each development domain, we used multiple logistic regression models to evaluate each outcome variable separately. Six KIDS subscales were recorded as binary variables so that “1” indicated passing, and “0” not passing, of the developmental norms for each domain. According to the KIDS manual, approximately 65 percent of the general population reach these developmental norms.

Included in the model as independent variables were infant temperament and family environment factors including maternal education level (>high school or ≤high school), type of family (nuclear or extended), household income (>4 million or ≤4 million yen per year), maternal cognitive stimulation (high or low), coparenting behavior (high or low), and maternal parenting stress (high or low). The confounding factors considered in the multiple logistic regression analyses were birth weight (low or normal), gestational age (preterm or full-term), birth order (first or other), and temperament. Odds ratios and 95% confidence intervals were calculated. All analyses in this study were performed using SPSS Version 15.

### Ethics

This study protocol was approved by the Ethical Review Committee of the collaborating research institutes (Osaka City General Hospital, Mie-chuo Medical Center and Faculty of Regional Sciences, Tottori University) and the Ethical Review Committee of the Research Institute of Science and Technology for Society, Japan Science and Technology Agency, which based their approval on the “Guidelines Concerning Epidemiological Research” promulgated by the Japanese Ministry of Education, Culture, Sports, Science and Technology and the Ministry of Health, Labour and Welfare.

## RESULTS

Approximately 76% of mothers had received some tertiary education and 70% of families had an income of at least 4 million Japanese yen per year. In this sample, approximately 7% of infants had a low birth weight and 3% were preterm. The range of birth weights was 1524 to 4066 g and the gestational age range was 28 to 41 weeks. There were no significant differences in the distribution of characteristics between the 2 regions (Table 1). However, although there were no significant differences in infant sex, birth order, or the frequencies of prematurity or low birth weight, the characteristics of the participant mothers and their families differed from those of the general Japanese population: the sample included better-educated mothers, higher-income families, and a higher frequency of nuclear families. This may result in selection bias, ie, a higher percentage of mothers may have had a relatively affluent family background.

**Table 1.** Sociodemographic and birth characteristics (%) of the effective sample^a^ and the Japanese population

	Total (*n* = 270)	Osaka (*n* = 169)	Mie (*n* = 101)	Japan
Child sex				
​ Male	51.1	53.2	49.0	48.8^b^
​ Female	48.9	46.8	51.0	51.2
Birth order				
​ First	56.7	59.8	51.5	48.2^b^
​ Others	43.3	40.2	48.5	51.8
Birth weight				
​ ≥2500 g	92.9	93.5	92.2	90.5^b^
​ <2500 g	7.1	6.5	7.6	9.5
Gestational age				
​ ≥37 weeks	97.0	97.0	97.0	96.1^c^
​ <37 weeks	3.0	3.0	3.0	2.9
Type of family				
​ Nuclear	91.1	93.5	86.1	81.2^d^
​ Extended	8.9	6.5	13.9	18.8
Maternal education				
​ >high school	76.3	78.1	73.3	47.0^e^
​ ≤high school	23.7	21.9	26.9	53.0
Family income				
​ ≥4 million yen/year	70.4	72.8	66.3	79.1^f^
​ <4 million yen/year	29.6	27.2	33.7	20.9

^a^Among the 301 participants, 270 (effective sample) gave complete answers for all variables included in the logistic regression analyses.

^b^According to the reports of the Japan national census and population survey of 2005.

^c^According to the population survey of 2004.

^d^Among families that have children younger than 6 years, according to the national census of 2005.

^e^According to the national censes of 2000.

^f^Family income was limited in families with 2 workers and in those with a head of household younger than 40 years, according to the national survey of family income and expenditure of 2004.

The pass rates for developmental norms by domain were approximately 50% for physical/motor, 69% for manipulation, 55% for receptive language, 50% for expressive language, 62% for social relationships with adults, and 66% for feeding (Table 2). With regard to overall development, 68% of infants passed the developmental norm.

**Table 2.** Mean score, range, and pass rate for each developmental domain

Development domain	Mean (SD)	Range	Pass rate^a^
Physical/motor	16.5 (3.4)	9–25	50%
Manipulation	19.5 (2.7)	11–26	69%
Receptive language	8.6 (1.6)	2–13	55%
Expressive language	8.3 (2.0)	3–13	50%
Social relationships with adults	19.3 (3.2)	11–26	62%
Feeding	9.0 (2.1)	2–13	66%

^a^The cutoff points were set according to the manual of the Kinder Infant Development Scale Type A.

As shown in Table 3, children whose parents showed high levels of supportive coparenting developed significantly faster in manipulation, receptive language, and social skills. The pass rates for norms in receptive language, expressive language, social relationships, and feeding were significantly higher in children who received more stimulation from their mother or environment.

**Table 3.** Distributions of independent variables and their associations with the developmental status of 9-month-old infants

Variables	*n*	Developmental norm pass rates, by group (%)					

		Physical /motor	Manipulation	Receptive language	Expressive language	Social relationships	Feeding
Sex							
​ Male	138	52.9^a^	69.6	54.3	60.9	60.9	65.9
​ Female	132	40.9	69.7	54.5	68.2	68.2	62.9
Birth order							
​ First	153	47.1	68.6	52.9	53.6	62.7	65.4
​ Others	117	47.0	70.9	56.4	49.6	66.7	63.2
Birth weight							
​ ≧2500 g	251	48.2	71.7^a^	55.8	52.2	65.7^a^	66.1
​ <2500 g	19	31.6	42.1	36.8	47.4	47.4	42.1
Gestational age							
​ ≧37 weeks	262	48.1^a^	71.0^a^	55.3	51.9	75.0	64.9
​ <37 weeks	8	12.5	25.0	25.0	50.1	64.1	50.0
Type of family							
​ Nuclear	246	48.3^a^	69.2	52.9	47.7	61.2	65.2
​ Extended	24	65.0	67.5	65.0	55.0	70.0	72.5
Maternal education level							
​ >high school	206	47.1	68.9	53.3	52.9	65.5	66.0
​ ≦high school	64	46.9	71.9	48.4	48.4	60.9	59.4
Family income (per year)							
​ ≧4 million yen	190	45.8	69.5	57.4	51.1	64.2	63.2
​ <4 million yen	80	50.0	70.0	50.8	53.8	65.0	67.5
Coparenting^c^							
​ High	217	48.8	72.8^b^	57.1^a^	53.5	67.3^a^	63.1
​ Low	53	39.6	56.6	43.4	45.3	52.8	69.8
Maternal stimulation score^d^							
​ High	145	50.0	70.9	62.8^b^	59.3^b^	69.7^b^	71.7^b^
​ Low	125	50.8	67.0	44.8	43.2	58.4	56.0
Parenting stress at 4 months^d^							
​ High	156	45.5	67.6	67.9	57.1	51.9	66.0
​ Low	114	49.1	70.1	71.9	50.9	51.8	62.3
Parenting stress at 9 months^d^							
​ High	158	44.3	68.4	56.3	50.6	63.3	65.8
​ Low	112	50.9	71.4	51.8	53.6	66.1	62.5

^a^*P* < 0.05, ^b^*P* < 0.001. Analyses were performed using the chi-square test.

^c^High: Score of 1 or higher; Low: Score of 0.

^d^High: Score equal to median or higher; Low: Score lower than median.

There were positive and statistically significant correlations between scores for persistence in all development domains, except motor development (Table 4).

**Table 4.** Median child temperament scores and their correlation with development scores

Temperament	Median (range)	Physical /motor	Manipulation	Receptive language	Expressive language	Social relationships	Feeding
Frustration Tolerance	20 (6–36)	−0.127^a^	−0.106^a^	0.044	0.014	−0.056	−0.030
Persistence	22 (6–36)	0.022	0.131^a^	0.232^b^	0.210^b^	0.111^a^	0.168^b^
Approach	26 (6–36)	−0.087	−0.074	−0.114^a^	−0.056	−0.159^b^	−0.067
Rhythmicity	25 (6–36)	−0.030	0.014	0.104^a^	−0.001	0.029	−0.040

^a^*P* < 0.05,^ b^*P* < 0.01.

Table 5 shows the intercorrelations among independent variables. Among many statistically significant correlations, we noted 2 combinations of variables. First, maternal parenting stress scores at an infant age of 4 months were strongly positively correlated with scores at 9 months (*r* = 0.691, *P* < 0.01). Second, maternal parenting stress scores were negatively correlated with coparenting scores (*r* = −0.271, *P* < 0.01) and maternal cognitive stimulation (*r* = −0.129, *P* < 0.01).

**Table 5.** Bivariate correlations between selected independent variables

	Birth weight	Gestational age	Birth order	Education	Family income	Stress at 4 months	Stress at 9 months	Coparenting
Birth weight	1							
Gestational age	0.344^a^	1						
Birth order	0.049	−0.058	1					
Maternal education	−0.047	0.089	−0.024	1				
Family income	0.013	−0.039	0.041	0.253^a^	1			
Parenting stress at 4 months	0.056	−0.042	0.290^a^	−0.043	−0.074	1		
Parenting stress at 9 months	0.078	−0.044	0.188^a^	−0.017	−0.153^a^	0.691^a^	1	
Coparenting	0.060	0.121^a^	−0.062	0.128^a^	0.120^b^	−0.271^a^	−0.286^a^	1
Maternal stimulation	−0.033	0.019	−0.133^a^	0.030	0.023	−0.129^a^	−0.093	0.083

^a^*P* < 0.01, ^b^*P* < 0.05.

Table 6 summarizes the results of logistic regression analyses for 6 developmental outcomes. Factors such as birth weight and gestational age had significant effects on various domains of development. For example, low birth weight was negatively associated with manipulation, sociability, and feeding development. Preterm infants showed slower development in manipulation than did full-term infants. Girls showed slower motor development than did boys. After adjusting for these factors, perseverance in task situations remained associated with development in all domains except the physical/motor domain. Odds ratios suggest that infants who display greater persistence develop faster in almost every domain. Infants who tended to be receptive to new situations or people, however, displayed slower development in receptive language and sociability.

**Table 6.** Summary of factors significantly contributing to each domain of development in infants aged 9 months

Variables	OR^a^ (95% CI) for passing assessment					

	Physical /motor	Manipulation	Receptive language	Expressive language	Social relationships	Feeding
Child factors						
​ Sex (female)	0.51 (0.30–0.86)	ns	ns	ns	ns	ns
​ Birth order (first)	ns	ns	ns	ns	ns	ns
​ Birth weight (<2500 g)	ns	0.28 (0.10–0.83)	ns	ns	0.33 (0.11–0.94)	0.33 (0.12–0.96)
​ Gestational age (<37 weeks)	ns	0.13 (0.02–0.83)	ns	ns	ns	ns
​ Temperament						
​ ​ Frustration tolerance	0.94 (0.89–0.98)	ns	ns	ns	ns	
​ ​ Persistence	ns	1.06 (1.01–1.12)	1.12 (1.07–1.18)	1.08 (1.03–1.13)	1.05 (1.00–1.10)	1.07 (1.02–1.12)
​ ​ Approach	ns	ns	0.93 (0.89–0.97)	ns	0.94 (0.90–0.98)	ns
​ ​ Rhythmicity	ns	ns	ns	ns	ns	ns

Family factors						
​ Type of family (Nuclear)	ns	ns	ns	ns	ns	ns
​ Mother's education (≤high school)	ns	ns	ns	ns	ns	ns
​ Family income (≤4 million yen)	ns	ns	ns	ns	ns	ns
​ Coparenting (high)^b^	ns	2.27 (1.17–4.43)	1.94 (1.01–3.86)	ns	2.12 (1.07–4.03)	ns
​ Maternal stimulation (high)^c^	ns	ns	2.43 (1.39–4.25)	1.95 (1.16–3.20)	1.73 (1.00–3.00)	2.15 (1.24–3.72)
​ Parenting stress at 4 months (high)^c^	ns	ns	ns	ns	ns	ns
​ Parenting stress at 9 months (high)^c^	ns	ns	ns	ns	ns	ns

Abbreviations: ns, not significant.

^a^Odds ratios and 95% confidence intervals for passing score on an assessment of each development domain on multiple logistic regression analysis after adjustment for all variables.

^b^High: Score of 1 or higher; Low: Score of 0.

^c^High: Score equal to median or higher; Low: Score lower than median.

Cognitive stimulation was also found to be a significant contributor to development in domains related to sociability. When mothers scored high in cognitive stimulation, their children’s development in receptive language, expressive language, social relationships, and feeding was significantly faster, after controlling for other confounding factors. The ORs for 4 domains of development ranged from 1.96 to 2.43. Children whose parents scored high in supportive coparenting showed faster development in manipulation (OR, 2.27), receptive language (1.94), and social relationships (2.07).

## DISCUSSION

This study investigated parenting factors that contribute to several developmental domains of infants at 9 months of age. Overall, we found that mothers who interact with their children through a variety of cognitive stimulation, such as talking, singing, playing, and going out of the house together, play an important role in promoting their children’s linguistic and social development. Fathers who engaged in coparenting by communicating with their spouse about child care, or who participated in caring for the child during early infancy, independently contributed to their child’s development in manipulation, receptive language, and sociability, even after adjusting for the child’s birth weight and temperament.

It has been widely recognized that maternal teaching promotes cognitive growth in infants. Infants whose mothers respond sensitively to their social bids, vocalizations, and/or playing display greater competency in cognitive and language assessments.^21^^–^^24^ Our findings were consistent with those of previous studies, which showed that parental engagement in positive interactive activities with their children, including reading stories and playing together, promotes language development, as well as social and emotional development in young children.^24^^–^^26^ Through teaching, mothers who are sensitive and responsive to their child’s emotions, and who provide access to stimulating objects and encourage a child to progress just beyond their current level, may foster both persistence and advanced cognitive development in the future.^27^

Coparenting behavior is typically defined as the quality of coordination between adults in their parental roles^28^ and includes hostile, competitive, and supportive dimensions, as well as discrepancies in parental involvement.^29^ Our findings, which are consistent with previous results, suggest that high levels of supportive coparenting during infancy play an important role in promoting children’s social and cognitive development. Fathers who take part in caregiving activities usually discuss child care with their wives. When fathers and mothers agree on child rearing, fathers are more sensitive to their infants and may provide the needed support for overall family functioning. Although marital quality may influence a child’s development indirectly through its impact on parenting styles, it may also affect the child directly by creating a high level of tension at home or through the child’s internalization of the parents’ interaction styles.^30^ In this study, the level of coparenting was rated by mothers, which is not a sufficient measure to evaluate paternal involvement in child rearing. Studies with more direct measures are needed to explore how paternal involvement contributes to the several domains of development.

Surprisingly, we found no relationship between maternal parenting stress and any domain of child development. Generally, parenting stress is a risk factor for suboptimal parenting: it is associated with reduced maternal sensitivity and negative child outcomes.^10^^,^^31^ However, while many studies have examined the effects of maternal parenting stress on the development of toddlers and preschool children, few have focused on developmental outcomes during early infancy. Although the influence of maternal parenting stress on child development was not clear in this study at an infant age of 9 months, this influence might become apparent as children grow. In addition, in the present study, the scores for maternal parenting stress were negatively associated with those for coparenting and maternal stimulation, which suggests that maternal parenting stress influences parenting behavior and indirectly affects child development through the mediating variables of coparenting and stimulation.

Numerous studies have shown that poverty and a low level of parental education are associated with lower cognitive development.^7^^,^^32^^–^^34^ However, in the present study we failed to identify any salient socioeconomic factors—eg, type of family, maternal education level, and family income—on child development. These results may be due to the generally higher educational level of mothers and the narrower range of family income in this sample, as compared to the general population. Our results do support previous work that showed an association between birth weight and child development,^2^^,^^35^^,^^36^ and an association between temperament and cognitive/linguistic development.^37^^,^^38^ Tamis-LeMonda et al provided evidence that the attention processes may be particularly important in language acquisition.^39^ In contrast to children who display persistence, those with a receptive temperament displayed slower development in language comprehension and social relationships with adults. A review of the literature indicates that an infant’s negative reaction to approaching strangers commonly appears by 8 or 9 months of age and increases through the first year.^40^ In our study, the results indicating that, at 9 months, children with low approachfulness had higher levels of development, as compared to children with greater approachfulness, might reflect a difference in the ability of infants to discriminate strangers.

Our main finding is that, after adjusting for child-related factors, parenting factors had a significant effect on child development in sociability. The main limitation of this study was that all measures relied on the reports of mothers, and thus the validity of these results is limited by the accuracy of parental responses. Another limitation is that because the assessment of child development outcomes in this study was made using a Japanese instrument, international comparison is impossible. In addition, this study was based in only a few areas of Japan, and the higher educational background of the participants indicates the possibility of selection bias in the sample. Therefore, we advise caution when applying our results to the general Japanese population. We hope to prevent participants from dropping out, so as to maintain the internal validity of our study.

Among the independent variables, child temperament and maternal stimulation behaviors were measured at the same time as the child developmental outcomes. Although simultaneous evaluation of variables and outcomes allows for identification of associations between them, it does not permit us to determine causality. Evaluation of a causal relationship between exposures and outcomes is valid only with variables measured before the outcome. Additional longitudinal data analyses are needed to investigate causal relationships in these variables. Nevertheless, we believe that these findings provide a promising direction for future research on maternal parenting behaviors and child development.

The strength of this study is that it is the first to identify factors that positively contribute to multiple domains of development in infancy in Japan. To cite an example, we found that maternal cognitive stimulation was consistently associated with the developmental domains related to sociability, but not with other domains. Anme and Segal found that development of communication ability and intelligence among children was significantly related to social stimulation.^14^ Our study examined a wider range of explanatory variables and found that these variables had significant effects on a broader range of domains. The results from these two studies are similar in that they both highlight the importance of variety in daily stimulation for the development of social skills in children. This finding might have implications for future intervention studies on the promotion of child social development. The other strength of this study is that the analysis was controlled for a wide range of potential confounders, including birth weight, gestational age, temperament, maternal education, and socioeconomic status. In the future, we hope to further analyze these data to elucidate the relationship between multiple outcome variables by, for example, making use of a latent variable path analytic approach. Such an attempt was reported by Yamagata and the JCS Group.^41^

This study has contributed to current knowledge by describing the parenting factors that have an impact on several domains of development in infancy. Our results show that early coparenting behavior and maternal cognitive stimulation contributed to every sociability-related domain of development, even after adjustment for possible confounding factors. Given these results, we believe it is important to develop preventive or psychoeducational interventions that are focused on helping young parents to establish and maintain successful coparenting relationships and cognitive stimulation behaviors as their children grow.

More information is needed to clarify the long-term relationships between the factors explored in the present study and the several domains of development. These are the issues on which we will focus in subsequent stages of our longitudinal study.

## APPENDIX

### Japan children’s study group

Chairman: Zentaro Yamagata (Department of Health Sciences, School of Medicine, University of Yamanashi), Hideaki Koizumi (Advanced Research Laboratory, Hitachi, Ltd.).

Participating researchers: Yoko Anji, Yuka Shiotani, Mizue Iwasaki, Aya Kutsuki, Misa Kuroki, Naho Ichikawa, Tomoyo Morita, Haruka Koike, Yusuke Morito, Shunyue Cheng, Hiraku Ishida, Hisakazu Yanaka, Daisuke Tanaka, Kumiko Namba, Tamami Fukushi, Hiroshi Toyoda, Shihoko Kimura-Ohba, Akiko Sawada (Research Institute of Science and Technology for Society, Japan Science and Technology Agency), Kevin K. F. Wong (Department of Anesthesia and Critical Care, Massachusetts General Hospital), Yoichi Sakakihara (Department of Child Care and Education, Ochanomizu University), Hideo Kawaguchi (Advanced Research Laboratory, Hitachi, Ltd.), Toyojiro Matsuishi (Department of Pediatrics and Child Health, Kurume University), Shunya Sogon (The Graduate Division of the Faculty of Human Relations, Kyoto Koka Women's University), Kiyotaka Tomiwa, Tomonari Awaya, Sigeyuki Matuzawa (Graduate School of Medicine, Kyoto University), Shoji Itakura (Graduate School of Letters, Kyoto University), Masako Okada (Koka City Educational Research Center), Yoshihiro Komada (Department of Pediatric and Developmental Science, Mie University Graduate School of Medicine, Institute of Molecular and Experimental Medicine), Hatsumi Yamamoto, Noriko Yamakawa, Motoki Bonno (Clinical Research Institute, Mie-chuo Medical Center, National Hospital Organization), Mariko Y. Momoi, Takanori Yamagata, Hirosato Shiokawa (Department of Pediatrics, Jichi Medical University), Norihiro Sadato, Daisuke N Saito (National Institute for Physiological Sciences, National Institutes of Natural Sciences), Hitoshi Uchiyama (Matsue Co-medical College), Tadahiko Maeda, Tohru Ozaki (The Institute of Statistical Mathematics, Research Organization of Information and Systems), Tamiko Ogura (Graduate School of Humanities, Kobe University), Hiroko Ikeda (National Epilepsy Center Shizuoka Institute of Epilepsy and Neurological Disorder), Koichi Negayama (Graduate School of Human Sciences, Waseda University), Kayako Nakagawa (Graduate School of Engineering, Osaka University), Kanehisa Morimoto (Graduate School of Medicine, Osaka University), Tokie Anme (Graduate School of Comprehensive Human Sciences, University of Tsukuba), Katsutoshi Kobayashi (Center for Education and Society, Tottori University), Tatsuya Koeda, Toshitaka Tamaru, Ayumi Seki, Shinako Terakawa, Ariko Takeuchi (Faculty of Regional Sciences, Tottori University), Yukuo Konishi (Department of Infants’ Brain & Cognitive Development, Tokyo Women’s Medical University), Osamu Sakura (Interfaculty Initiative in Information Studies, The University of Tokyo), Masatoshi Kawai (Institute for Education, Mukogawa Women's University), Sonoko Egami (Hokkaido University of Education), Takahiro Hoshino (Graduate School of Economics, Nagoya University), and Yuko Yato (College of Letters, Ritsumeikan University).
